# Interleukin-17A expression in patients presenting with nasal polyposis

**DOI:** 10.5935/1808-8694.20130110

**Published:** 2015-10-08

**Authors:** Melissa Ameloti Gomes Avelino, Isabela Jubé Wastowski, Ricardo Gimenes Ferri, Thaís Gomes Abrahão Elias, Ana Paula Lindoso Lima, Larissa Mesquita Nunes, Shirley Shizue Nagata Pignatari

**Affiliations:** a PhD; Adjunct Professor - Federal University of Goiás.; b PhD; Professor - State University of Goiás.; c MSc; Professor - Pontifical Catholic University of Goiás.; d Medical Student - Pontifical Catholic University of Goiás.; e Medical Student - Pontifical Catholic University of Goiás.; f MD; PhD; Professor - Federal University of São Paulo. Federal University of São Paulo.

**Keywords:** allergy and immunology, interleukin-17, nasal polyps

## Abstract

Sinonasal polyposis (SNP) is a chronic inflammatory pathology of the nasal/paranasal cavities which affects from 1%-4% of the population. Although polyps seem to be a manifestation of chronic inflammation of nasal/paranasal sinus mucosa in both allergic and non-allergic subjects, the pathogenesis of nasal polyposis remains unknown. Interleukin-17A (IL-17A) is a key inflammatory cytokine in many disorders. Little attention has been paid to the role of IL-17A in chronic inflammatory disorders.

**Objective:**

To investigate the expression of IL-17A in the SNP and verify if this expression is a marker of good or bad prognosis.

**Method:**

Prospective study with 25 patients presenting with SNP were subjected to the immunohistochemistry technique. After a skin prick test, all patients were divided into atopic and nonatopic groups, and asthmatic or non-asthmatic.

**Results:**

The IL-17A expression was observed in both atopic and nonatopic patients. The numbers of IL-17A positive cells were greater in nasal polyps of atopic patients than nonatopic (*p* = 0.0128).

**Conclusion:**

These results indicate that IL-17A may play an important role in the pathology of SNP. Considering the inflammatory properties of IL-17A, this study suggests that it could increase susceptibility to atopy and asthma.

## INTRODUCTION

Sinonasal polyposis (SNP) or chronic rhinosinusitis with nasal polyps is a chronic inflammatory pathology of the nasal and paranasal cavities^1^, ^2^, which affects from 1% to 4% of the population and has a clear association with asthma, aspirin sensitivity and cystic fibrosis^3^.

Patients with SNP typically present with nasal obstruction, rhinorrhea, hyposmia and reduced quality of life^3^, ^4^. Although polyps seem to be a manifestation of the chronic inflammation of nasal/paranasal sinus mucosa in both allergic and non-allergic subjects, the pathogenesis of nasal polyposis remains unknown^5^, ^6^, but it is probably a multifactorial disease with several different etiological factors, and chronic persistent inflammation is undoubtedly a major factor irrespective of the etiology^5^. Chronic inflammation of the mucous represents a challenge for the otolaryngologist.

The diagnosis of SNP is confirmed by nasal endoscopy or a computed tomography (CT) scan^1^. Despite the major impact on quality of life^7^, in the literature there are no concerns about biomarkers involved in the pathogenesis of nasal polyps and their possible contributions to the prognosis of SNP.

For more than two decades, immunologists have been using the so-called Th1/Th2 paradigm to explain most of the phenomena related to adaptive immunity. The Th17 cells, was recently described as a distinct lineage that does not share developmental pathways with either Th1 or Th2 cells. Shen et al. suggested that the imbalance of Treg/Th17 may play an important role in the development of SNP and that atopy may aggravate SNP^8^. Shen et al., on another study, suggested an important part of IL-17A in SNP, and demonstrated that expression of IL-17A was significantly upregulated in SNP patients and was more severe in atopic SNP^9^.

Little attention has been paid to the role of IL-17A in autoimmune and chronic inflammatory disorders, but evidence shows that the expression of IL-17A in chronic inflammatory skin diseases including psoriasis and atopic dermatitis^10^ is associated with a worst clinical course of the disease^10^, ^11^.

Considering this lack of understanding of the mechanism which triggers inflammation and which hinders the development of new treatments for this disease, and the importance of the characterization of inflammatory mediators involved in the pathogenesis of SNP, the objective of this study was to investigate the IL-17A expression in SNP and verify if this expression is a marker of worse prognosis.

## METHOD

### Study population

This was a longitudinal contemporary cohort in which twenty-five patients, who presented with sinonasal polyposis and who had been submitted to surgery for polyp resection, were evaluated. Patients without sinonasal polyposis identified in the computed tomography (CT) scan and nasal endoscopy were excluded from this study. All underwent clinical otorhinolaryngol evaluation with a nasal endoscopy, CT-scan, lung function test (spirometry) and the skin prick test before surgery. The preoperative CT scans were graded according to the Kennedy classification, to evaluate the extent of patients with SNP^10^. And all were investigated for allergy and positive history for asthma. They were divided in two groups: atopic (positive skin prick test) and non-atopic patients (negative skin prick test). The skin prick test was performed with 10 extracts: histamine (positive control), saline solution (negative control), *D. pteronyssinus, D. farinae, Blomia tropicalis, Felix domesticus, Canis familiars, P. Americana, Aspergillus fumigatus and Alternaria alternate*. The response was considered positive when there was a halo of 3 mm larger than that of the negative control.

The SNP patients were classified as having positive or negative history for asthma, which was confirmed by spirometry. This study was approved by the Research Ethics Committee, under protocol 0711/11 approved in 06/02/11. All patients signed a term of informed consent for participation in the study.

### Immunohistochemistry

After resection surgery, the polyps were subjected to the immunohistochemistry technique. Four-micrometer sections were cut from paraffin-embedded specimens. The Universal HRP-Polymer MACH 4 detection system (Biocare Medical, Concord, CA, USA) was used. In summary, after rinsing the sections in phosphate buffered saline with 0.1% saponin, endogenous peroxidases were inhibited using H_2_O_2_. Samples were initially incubated with specific or irrelevant antibodies for 1 hour at room temperature and subsequently with a solution containing a MACH 4 Mouse Probe for 15 minutes. Diaminobenzidine plus a chromogen-substrate was used to develop antibody fixation. The specific monoclonal antibodies MEM-G/2 (Exbio, Praha, Czech Republic) recognize the free heavy-chain of anti-IL-17 (Santa Cruz Biotechnology, Santa Cruz, California, USA). An identical IgG1 isotype anti-desmin antibody which was run simultaneously with each sample served as a negative control.

### Evaluation of stained sections

The immunohistochemical analysis was carried out on polyp tissue. Immunoreactivity was scored using a semi-quantitative scoring method by evaluating the percentage of positive cells. The cut-off scores for determining the positivity of IL-17A detected by immunohistochemistry were obtained by the receiver operating characteristic (ROC) curve analysis. ROC curve analysis was performed for IL-17A expression. All sections were blindly analyzed using a light microscope with high-power fields (400x).

### Statistical analysis

The ROC curve of staining performance for the determination of IL-17A cut-off expression was performed. The immunostaining scores were compared with the Mann-Whitney U test and the correlations of the immunostaining scores were tested with Spearman correlation analysis. Comparative analyses between the groups were performed by the two-sided Fisher exact test. A *p*-value of less than 0.05 was considered significant. All statistical analyses were performed using the GraphPad Instat (version 5.0).

## RESULTS

### Clinical and Epidemiological Findings

The results included 25 patients with sinonasal polyposis submitted to nasal endoscopy and CT-scan to confirm the disease. The patient group consisted of 13 (52%) males and 12 (48%) females aged between 35 and 83 (mean: 48.8 years). The patients were divided into two groups: the group with a negative prick test (non-atopic group) consisted of 13 patients and the other group consisted of 12 patients with positive prick test (atopic group). Of these patients, 17 presented asthma.

**Graph 1:**
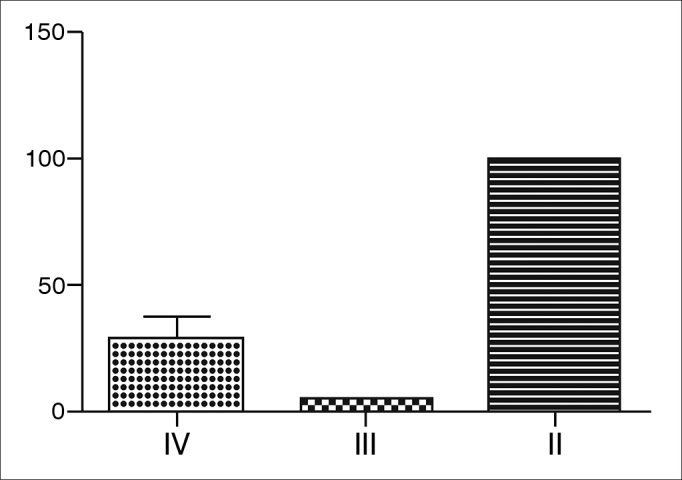
Kennedy classification of patients with SNP - Most patients were classified as grade II, namely, most patients had bilateral ethmoid disease, involving a paranasal sinus.

Using CT-scan evaluation, patients were classified according to the Kennedy et al. criteria to evaluate the extent of SNP (Graph 1). Most patients were classified as grade II.

### The expression of IL-17A

The IL-17A expression was observed in epithelial and mainly by inflammatory infiltrating cells.

On ROC curve analysis, the cut-off for positive results was 50% or greater (AUC = 0.892; *p* < 0.0001, 95% CI 0.665 to 0.986) considering that, IL-17A could be detected in 12 out the 19 specimens evaluated (63.1%).

**Graph 2:**
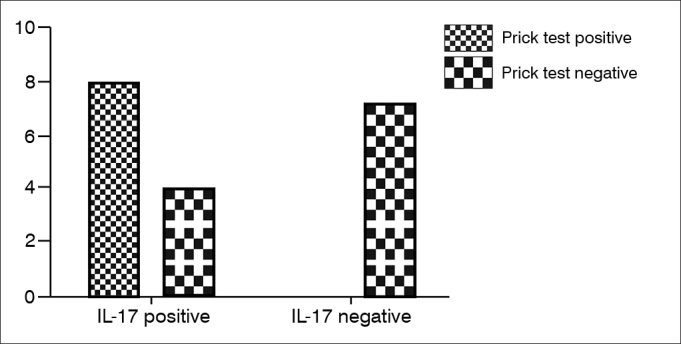
Expression of IL-17 and prick test result - The number of IL-17 positive cells were greater in nasal polyps of atopic patients than non-atopic.

The number of IL-17A positive cells were greater in nasal polyps of atopic patients than non-atopic (*p* = 0.0128, RR = 0.33, 95% CI: 0.1497 to 0.7421). However, no statically significant result was found between asthmatic and non-asthmatic patients (*p* > 0.05) (Graph 2).

## DISCUSSION

SNP is a chronic inflammatory condition associated with substantial impaired quality of life, reduced workplace productivity, and considerable medical treatment costs^7^, ^12^. Despite the fact that this recent research evidence contributes to further comprehension of the pathophysiology of this chronic airway condition, the pathogenesis of SNP remains poorly understood and appears to be multifactorial, being associated with conditions such as atopy, asthma, cystic fibrosis, aspirin sensitivity and chronic rhinosinusitis^13^, ^14^, ^15^.

A diverse spectrum of alterations involving T-cell patterns, cytokine profiles, IgE production, microorganisms and immune system malfunctions should be associated with SNP pathogenesis^16^, ^17^. In this context, this study evaluated the expression IL-17A in SNP.

This analysis showed that the IL-17A was expressed in 12 patients with SNP. Interestingly, the IL-17A expression tended to be significantly more frequent in patients with positive prick test, or in other words, atopic patients (*p* = 0.0128).

Agreeing with this study, experimental model showed that the absence of IL-17 in mice compromises the development of contact hypersensitivity reaction, reinforcing the importance of these cells in contact sensitivity^18^. Studies suggest that IL-17A produced by cells are induction of proinflammatory cytokines (such as IL-1, IL-6 and TNF-α), chemokines (CXCL1, CXCL2, CXCL5 and CXCL8) and adhesion molecules (ICAM-1 and VCAM-1) by epithelial and endothelial cells, thus leading to the recruitment of inflammatory cells and interaction of these cells with the epithelium^19^. This way, IL-17 increases the local inflammatory process^20^.

Based on the results of this study it can be speculated that the expression of IL-17A in SNP may contribute to increase susceptibility to atopy in SNP, a factor that aggravates the disease.

## CONCLUSION

The results of this study indicated that IL-17A may play an important role in the pathology of sinonasal polyposis. After considering the inflammatory properties of IL-17A, these authors suggest that IL-17A could increase susceptibility to atopy and SNP.
